# Complications After Laparoscopic and Conventional Cholecystectomy: A Comparative Study

**DOI:** 10.1155/1994/59865

**Published:** 1994

**Authors:** Iris B. Brune, K. Schönleben, S. Omran

**Affiliations:** Chirurgische Klinik Klinikum Ludwigshafen Germany

## Abstract

The growing popularity of laparoscopic cholecystectomy (LC) has made extensive series comparing laparoscopic and conventional cholecystectomy in a prospective, randomized way nearly impossible. To evaluate LC we compared retrospectively 800 laparoscopic with 748 conventional cholecystectomies (CC). Of the 800 LC, 10 (1.2%) were converted to laparotomy. 6 conversions were related to aberrant anatomical features or features making dissection very difficult, 4 conversions were due to complications. There were 5 (0, 6%) intraoperative complications during LC and 4 (0.5%) during CC. Postoperative morbidity was 2.1% (*n* = 17) after LC and 3.7% (*n* = 28) after CC. Particularly the incidence of wound problems was only 0.5% (*n* = 4) after LC while it was 1.3% (*n* = 10) after CC. Overall morbidity was 2.7% (*n* = 22) for LC and 4.2% (*n* = 32) for CC. Mortality rate after CC was 0.4% (*n* = 3), there were no deaths after LC. Common bile duct-injury rate was 0.2% (*n* = 2) for both groups. Complication rates after LC have been rapidly decreasing with growing experience. Laparoscopic cholecystectomy can safely be performed by appropriately trained surgeons in more than 90% of patients suffering from gallbladder disease. The low morbidity and mortality together with the significant advantages to patient recovery makes laparoscopic cholecystectomy the treatment of choice for symptomatic cholecystolithiasis.


*HPB Surgery*, 1994, Vol. 8, pp. 19–25
Reprints available directly from the publisher
Photocopying permitted by license only

© 1994 Harwood Academic Publishers GmbH
Printed in Malaysia

### Complications After Laparoscopic and Conventional Cholecystectomy: A Comparative Study

IRIS B. BRUNE, K. SCHÖNLEBEN and S. OMRAN

Chirurgische Klinik, Klinikum Ludwigshafen Germany

The growing popularity of laparoscopic cholecystectomy (LC) has made extensive series comparing laparoscopic and conventional cholecystectomy in a prospective, randomized way nearly impossible. To evaluate LC we compared retrospectively 800 laparoscopic with 748 conventional cholecystectomies (CC). Of the 800 LC, 10 (1.2%) were converted to laparotomy. 6 conversions were related to aberrant anatomical features or features making dissection very difficult, 4 conversions were due to complications. There were 5 (0, 6%) intraoperative complications during LC and 4 (0.5%) during CC. Postoperative morbidity was 2.1% ($n = 17$) after LC and 3.7% ($n = 28$) after CC. Particularly the incidence of wound problems was only 0.5% ($n = 4$) after LC while it was 1.3% ($n = 10$) after CC. Overall morbidity was 2.7% ($n = 22$) for LC and 4.2% ($n = 32$) for CC. Mortality rate after CC was 0.4% ($n = 3$), there were no deaths after LC. Common bile duct-injury rate was 0.2% ($n = 2$) for both groups. Complication rates after LC have been rapidly decreasing with growing experience. Laparoscopic cholecystectomy can safely be performed by appropriately trained surgeons in more than 90% of patients suffering from gallbladder disease. The low morbidity and mortality together with the significant advantages to patient recovery makes laparoscopic cholecystectomy the treatment of choice for symptomatic cholecystolithiasis.

KEY WORDS: Laparoscopic cholecystectomy Peritonescopy Cholangiography adverse effect

### INTRODUCTION

Since Carl Langenbuch performed the first successfull cholecystectomy in 1882 this operation was considered the "Gold Standard" in the treatment of symptomatic cholelithiasis[1–3] associated with minimal risk to the patient and a high degree of relief from symptoms.

Several years ago, a new technique which is less invasive and just as effective emerged: Mühe (Germany, 1986) and Mouret (France, 1987) were the first to perform laparoscopic cholecystectomy. Since then, due to growing experience and development of specially adapted laparoscopic instruments the technique has improved immensely.

The obvious advantages for the patient like a substantial reduction of hospitalization and recovery period, improved cosmesis and minimized postoperative pain[4,5] together with increased publicity by the media made laparoscopic cholecystectomy very popular within a short time. The new method spread rapidly throughout the surgical world.

However, before considering laparoscopic cholecystectomy to be the method of choice in the treatment of cholelithiasis, its results have to be compared to those of the conventional operation. The growing popularity of laparoscopic cholecystectomy has made extensive series comparing laparoscopic with conventional cholecystectomy in a prospective, randomised way nearly impossible in Germany. For this reason we compared, retrospectively, 800 laparoscopic with 748 conventional elective, consecutive cholecystectomies performed in our service.

### MATERIALS AND METHODS

From March 1983 to February 1990, 748 patients (group 2) underwent conventional cholecystectomy for symptomatic gallbladder disease at the Surgical Department, Klinikum Ludwigshafen. In March 1990, the first laparoscopic cholecystectomy was performed. As of July 1992, laparoscopic cholecystectomy had been performed on 800 patients (group 1).

The indication was symptomatic cholelithiasis for all patients. Patients with common duct stones were excluded from group 2. Preoperative evidence of common duct stones that could not be removed endoscopically was considered to be a contraindication for laparoscopic cholecystectomy. In the beginning we considered previous upper abdominal surgery, acute cholecystitis or a bilio-digestive fistula to be contraindications to laparoscopic surgery. For the last two years however, the only remaining contraindication were common duct stones and the suspicion of gallbladder carcinoma. Therefore group 1 was more selective than group 2.

Preoperative work-up included for both groups routine history, physical examination, laboratory testing and ultrasonographic evaluation of the gallbladder. All patients were preoperatively referred for either endoscopic or contrast radiographic evaluation of the upper gastrointestinal tract.

In addition to this, all patients in the laparoscopic group underwent preoperative i.v. cholangiography to exclude common bile duct stones or identify anomalous biliary ductal anatomy since we only started performing intraoperative, laparoscopic cholangiography routinely in February 1992. In the early phase of laparoscopic cholecystectomy intraoperative cholangiography was very tedious but with growing experience, it could be done as quickly as open surgery and it became part of the routine operative schedule. Routine intraoperative cholangiography was part of all conventional cholecystectomies.

Postoperatively, a regular diet was assumed on the next morning for group 1. To the patients in group 2, liquids were provided on the first postoperative day and a regular meal on the second day. Postoperative follow-up included laboratory studies and an abdominal sonogram on day 1 and 3. Most patients in group 1 were discharged from the hospital on the third postoperative day. After conventional cholecystectomy, patients were usually discharged on the 8th day after an uneventful postoperative course.

Complications were defined as any problem that delayed discharge, resulted in an undesired change in therapy, in readmission to the hospital or was a recognized complication of cholecystectomy such as biliary injury or leakage, hemorrhage or infection 6.
Among complications, we distinguished three groups:

— *minor controlable intraoperative problems:*
Events like gallbladder perforation, loss of stones, cystic artery bleeding or hemorrhage from small liver tears occurred in both patient groups, but since they were controlable and had no consequences for the subsequent operative course, they had not been recorded precisely for group 2.
— *major intraoperative complications:*
These complications resulted in an undesired change in therapy such as the conversion of laparoscopic to conventional cholecystectomy or repair of a bile duct injury, for example.
— *postoperative complications:*
These required postoperative medical therapy, a relaparoscopy or even a laparotomy.

### RESULTS

Of the 800 laparoscopically operated patients, 75% (599) were female and 25% (201) male. For the conventional group, this ratio was 72% (539) and 28% (209). The ages ranged from 14 to 91 years with an average of 50 years in group 1 and from 17 to 94 (average 59 years) in group 2. The leading symptoms were right upper abdominal pain (78% for group 1 and 67% for group 2), colic (53% and 49%), nausea (16% and 13%), vomiting (13% and 12%) and jaundice (7% and 5%).

Comparison of previously performed, abdominal operations showed no significant differences among the two groups for the number and spectrum of operations (Table 1).

Minor, controlable intraoperative complications occurred in 24% of laparoscopic cholecystectomies (Table 2). Since they did not result in a change of therapy or delay discharge, they were not considered to be relevant for complication rates.

We observed five major complications in group 1 and four in group 2 (Table 3 and 4). The frequency of common bile duct-injury was 0.2% in both groups. The two bile duct-lesions in group 2 were recognized intraoperatively and repaired immediately. In both patients of which one had acute cholecystitis, a small perforation of the CBD occurred during dissection of the junction between cystic duct and CBD. Both were identified before cholangiography. They were repaired

**Table 1** Previously performed operations before 800 laparoscopic (Group 1) and 748 conventional (Group 2) cholecystectomies.

| *Operation* | *Group 1* *n* | % | *Group 2* *n* | % |
|---|---|---|---|---|
| Appendectomy | 241 | 30% | 229 | 30% |
| Hysterectomy | 172 | 21% | 166 | 22% |
| Ovarectomy | 48 | 6% | 48 | 6% |
| Bowel resection | 4 | 0.5% | 3 | 0.4% |
| Others | 119 | 15% | 130 | 17% |
| Total of previously operated patients | 415 | 52% | 379 | 51% |

**Table 2** Controlable, intraoperative problems during 800 laparoscopic cholecystectomies.

| | *n* = 190/800 | (24%) |
|---|---|---|
| • | Gallbladder perforation | 130 |
| • | Loss of stones | 36 |
| • | Cystic artery bleeding | 46 |
| • | Liver bleeding | 23 |

**Table 3** Intraoperative complications during 800 laparoscopic cholecystectomies.

| *Lesion of* | *n* | % |
|---|---|---|
| Common bile duct | 2 | 0.2% |
| Small intestine | 2 | 0.2% |
| Aorta | 1 | 0.1% |
| Total | 5 | 0.6% |

**Table 4** Intraoperative complications during 748 conventional cholecystectomies.

| *Lesion of* | *n* | % |
|---|---|---|
| Common bile duct | 2 | 0.2% |
| Duodenum | 1 | 0.1% |
| Liver | 1 | 0.1% |
| Total | 4 | 0.5% |

by suture and T-tube placement. The patients have been submitted to ERCP 13 and 18 months respectively postoperatively: there was no evidence of CBD stricture or stenosis.

In group 1, a total transsection of the common bile duct that had been dissected and mistaken for the cystic duct was only recognized on the first postoperative day, when the patient had abnormal results on liver-function tests and abdominal pain. This patient required a second operation and Roux-en-Y hepaticojejunostomy. At reevaluation 17 months postoperatively, the patient was asymptomatic, all laboratory findings were within the normal range. This complication could have been avoided by intraoperative cholangiography, which we did not perform at that time. In the second patient, a tangential lesion was inflicted on the CBD during dissection of the gallbladder whose infundibulum was adherent to the CBD. It was discovered before cholangiography. After conversion to laparotomy, a T-tube was placed and the lesion repaired. Clinical and laboratory follow-up has been uneventful for 11 months.

In group 1, the only procedure-related, intraoperative complication was an aortic injury caused by the initial insertion of a trocar without peritoneal visualisation. After an immediate laparotomy, the injury was repaired and cholecystectomy finished conventionally. The patient, a 28 year old woman, was discharged on the 8th postoperative day without further complications.

Laparoscopic cholecystectomy was successfull in 790 of 800 attempts. 10 patients (1.2%) underwent conversion from laparoscopic to conventional cholecystectomy (Table 5). In 6 patients (0.75%) the reason for conversion was related to aberrant anatomical features or features making dissection extremely difficult. In 4 patients (0.5%), conversion was due to complications. There was no conversion associated with technical problems.

Postoperative complication rates were 2.1% ($n = 17$) after laparoscopic and 3.7% ($n = 28$) after conventional cholecystectomy (Tables 6 and 7). Procedure related complication rates were 2.0% ($n = 16$) in group 1 and 3.0% ($n = 23$) in group 2. Wound problems occurred in

**Table 5** Reasons for conversion of laparoscopic to conventional cholecystectomy in 800 patients.

| | *n* = 10/800 (1.2%) |
|---|---|
| *"Switching" for anatomic reasons* | |
| 2 | Adhesions |
| 1 | Intrahepatic gallbladder |
| 1 | Bilio-intestinal fistula |
| 1 | Carcinoma |
| 1 | Anatomic uncertainty |
| *Complications* | |
| 1 | Aortic injury |
| 1 | Common bile duct injury |
| 2 | Small intestine injuries |

4 patients (0.5%) of group 1 and in 10 patients (1.3%) of group 2. They were less severe in group 1 due to the small size of the incisions.

The frequency of cystic duct leaks was 0.1% in both groups. Both patients were treated with percutaneous drainage and endoscopic placement of biliary stents.

A common bile duct stenosis occurred in group 1: at the fifth laparoscopic cholecystectomy, concern about

**Table 6** Postoperative complications and treatment after 800 laparoscopic cholecystectomies.

| *Complication* | *n* | *Treatment* |
|---|---|---|
| *Surgical complications* | 16 (2.0%) | |
| Wound hematoma | 2 | none |
| Wound infection | 1 | local treatment |
| Hernia | 1 | hernia repair |
| Pancreatitis | 1 | medical |
| Cystic-duct leak | 1 | endosc. drainage |
| Intraabdom. abscess | 1 | laparotomy |
| CBD stone | 2 | endoscopy |
| CBD stenosis | 1 | endosc. stent |
| Subhep. bile collection | 2 | spontan stopped |
| Hemorrhage | 4 | 2 laparotomies<br>2 relaparoscopies |
| *Non-specific complications* | 1 (0.1%) | |
| Thrombosis | 1 | i.v. heparin |
| Total | 17 (2.1%) | |

**Table 7** Postoperative complications and treatment after 748 conventional cholecystectomies.

| *Complication* | *n* | *Treatment* |
|---|---|---|
| *Surgical complications* | 23 (3.0%) | |
| Wound hematoma | 2 | none |
| Wound infection | 6 | local treatment |
| Evisceration | 2 | laparotomy |
| Pancreatitis | 2 | medical |
| Cystic-duct leak | 1 | endosc. drainage |
| GI-hemorrhage | 1 | endoscopy |
| Intraabdom. abscess | 9 | 3 laparotomies<br>6 drainages |
| *Non-specific complications* | 5 (0.6%) | |
| Thrombosis | 1 | i.v. heparin |
| Pneumonia | 1 | medical |
| Emboly | 1 | lethal |
| Myocardial infarction | 1 | lethal |
| Renal insufficiency | 1 | lethal |
| Total | 28 (3.7%) | |

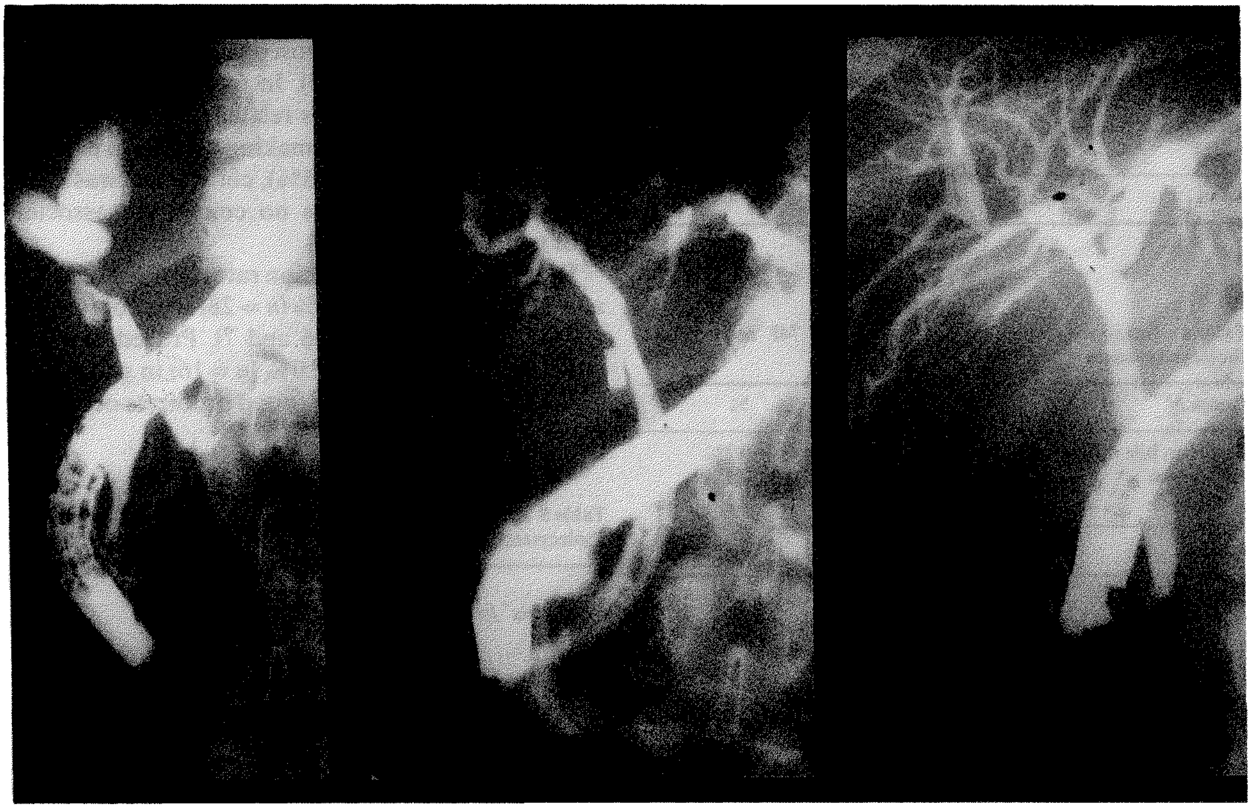

**Figure 1** Common bile duct stenosis after laparoscopic cholecystectomy.

**Table 8** Surgical reinterventions for complications after 800 lap. cholecystectomies.

| $n = 7/800$ (0.9%) | | | |
|---|---|---|---|
| | *Indication* | | *Operation* |
| 1 | Common bile duct injury | 1 | hepatico-jejunostomy |
| 1 | intraabd. abscess | 1 | laparotomy |
| 1 | hernia | 1 | hernia repair |
| 4 | hemorrhages | 2 | relaparoscopies |
| | | 2 | laparotomies |

**Table 9** Surgical reinterventions for complications after 748 Conventional cholecystectomies.

| $n = 5/748$ (0.6%) | | | |
|---|---|---|---|
| | *Indication* | | *Operation* |
| 2 | eviscerations | 2 | laparotomies |
| 9 | intraabdom. abscesses | 3 | laparotomies (6 drainages) |

leaving a long cystic duct stump made us place the Filshie-clip, used at that time, too close to the common bile duct thus catching its wall in the clip. ERCP on the 3rd postoperative day showed a stenosis that was treated by endoscopic biliary stent placement (Fig. 1).

In group 1, only one patient (0.1%) developed an intraabdominal abscess while this complication occurred in 9 cases (1.2%) in group 2.

Non-specific complications were reduced to one thrombosis (0.1%) in group 1. There were no deaths after laparoscopic cholecystectomy. After the conventional operation, the rate of non-specific complications was 0.6% ($n = 5$), mortality was 0.4% ($n = 3$).

Surgical reintervention for complications was necessary in 0.9% ($n = 7$) in group 1 (Table 8) and 0.6% ($n = 5$) in group 2 (Table 9).

### DISCUSSION

Laparoscopic cholecystectomy presents obvious advantages for the patient such as excellent cosmesis[4], shorter recovery periods[5] and improved postoperative pulmonary function[7]. Due to these advantages, laparoscopic cholecystectomy has, within the last two years, replaced conventional cholecystectomy and become the method of choice for the treatment of symptomatic gallbladder disease in our service.

The rapidly growing popularity of the new method made large, randomized studies comparing laparoscopic and conventional cholecystectomy impossible in Germany because most patients refused conventional cholecystectomy. To evaluate the laparoscopic operation, we retrospectively compared the results of our laparoscopic cholecystectomies with those of the conventional operation.

Operative time was much longer for laparoscopic cholecystectomies in the beginning but showed an impressive learning curve and decreased rapidly with growing experience. Operative duration varied considerably with the degree of difficulty of the operation. For simple cholecystectomies, it has become shorter laparoscopically than conventionally.

Intraoperative, cholangiography can be performed in about 90% of laparoscopic[4,8] and conventional[2] cholecystectomies. At present, there is no consensus in regard to the value, importance and safety of the routine use of intraoperative, laparoscopic cholangiography[8,9]. Our results show that it can safely be performed but there are reports about common bile duct injuries caused by cholangiography[5].

The aims of intraoperative cholangiography are to exclude common bile duct stones, injuries or anomalous bile duct anatomy. If an unsuspected common bile duct stone is identified during laparoscopic cholecystectomy, therapeutic options include laparoscopic common bile duct exploration, conversion to open cholecystectomy with common bile duct exploration or postoperative ERCP with stone extraction. Most authors prefer the last possibility[5,8] even though it is dependent on the availability of ERCP.

Although several techniques of laparoscopic common bile duct exploration using electrohydraulic or laser lithotripsy have already been described[10–14], these approaches require considerable dexterity and experience and, at the moment, add an important amount of time to laparoscopic cholecystectomy. This is why laparoscopic common bile duct exploration is not yet a routine practice but remains restricted to selected patients and very skilled, experienced surgeons. The expertise needed for these techniques can be acquired by routinely attempting cholangiography.

Of the 4 CBD injuries that occurred in both groups, only the total transsection of the CBD in group 1 might have been avoided by intraoperative cholangiography, which we had not been performing at the time. Mistaking the CBD for the cystic duct leads to most serious complications and seems to occur more frequently during laparoscopic operation. It can be avoided by intraoperative cholangiography. CBD injuries during conventional operation usually are less serious.

The controversial discussion about doing laparoscopic, intraoperative cholangiography routinely[9], in selected cases[4] or not at all is not concluded yet. We consider it to be an excellent training for the laparoscopic approach to the common bile duct but its results concerning the detection of CBD stones do not seem to be superior to those of preoperative cholangiography. The decision concerning the management of intraoperatively discovered common bile duct stones has to be taken individually in dependence of factors such as the patients age and general condition, size of the stones.

Initially, complication rates were very high after laparoscopic cholecystectomy but dropped with growing experience from 13.3% (operation 1–30) to 7.5% (31–70) and finally 2% (71–800). This is due to a learning effect and to the fact that laparoscopic cholecystectomy is only performed by laparoscopically experienced surgeons. It is not a routine teaching operation for residents while conventional cholecystectomy always was. In our service, conventional cholecystectomy had been mainly performed by the 18 residents (3rd–6th year). Laparoscopic cholecystectomy was done by a group of 6 surgeons of which only 3 were residents in training (4th–6th year). Our data clearly show the necessity of training programs and courses before performing laparoscopic surgery to avoid undue complications.

The reported, overall complication rates for laparoscopic cholecystectomy ranges between 2.7% and 5.1%[15,6,4]. After conventional cholecystectomy, 40–50% of specific complications were associated with wound problems. This has considerably improved for laparoscopic cholecystectomy. The incidence of wound problems has significantly decreased and the complications were less severe due to the small size of the incisions.

The rate of non-specific, postoperative complications was very low after laparoscopic cholecystectomy, there were no deaths. This was mainly due to the short recuperation time. However, the low mortality rate may well reflect the fact that the patients, at least in the beginning, were a select population undergoing elective surgery. Laparoscopic cholecystectomy is minimally invasive but must be considered a major surgical procedure performed under general anesthesia and thus carrying with it risks similar to those of conventional cholecystectomy.

Initially, the incidence of common bile duct injuries was reported to be higher for laparoscopic than for conventional cholecystectomy[6,16,12]. Lack of experience with the new technique seems to have been responsible for this. With growing experience, common bile duct injuries become less frequent, their incidence is not higher for the laparoscopic technique any more, even though they seem to be more severe. Comparison between our two groups is difficult due to the fact that intraoperative cholangiography had not been performed for group 1 in the first year. To avoid common bile duct injuries, it is important to expose the cystic-common duct junction in all cases and begin the dissection of the cystic duct at the infundibulum of the gallbladder[12]. In doubtfull cases, intraoperative cholangiography helps to define the anatomy and should allow all experienced surgeons to perform the operation without untoward morbidity from common duct injuries. Unknown as yet is the incidence of late biliary stricture and of problems related to long cystic duct stumps.

In 162 patients undergoing laparoscopic cholecystectomy, we encountered unexpected signs of disease such as acute cholecystitis, hydrops or empyema of the gallbladder. Since we could manage all these situations laparoscopically, they are no longer considered to be contraindications for laparoscopic cholecystectomy. The applicability of the new method has now reached 98% in our institution: only 2% of our patients presented with common bile duct stones that could not be extracted endoscopically and were therefore submitted to conventional cholecystectomy with common bile duct exploration.

In other series, conversion rates of 4, 7%[6], 5%[4], 6, 9%[5] or 8%[15] have been reported. In our service the rate of conversion from laparoscopic to conventional cholecystectomy was rather low with 1.2%. This is mainly due to the precise preoperative assessment of the common bile duct: there was no coincidental, intraoperative finding of common bile duct stones, most of the relevant biliary ductal abnormalities had been recognized on the preoperative i.v. cholangiogram.

Laparoscopic cholecystectomy can be performed safely in more than 90% of all patients suffering from symptomatic gallbladder disease. It is less invasive than conventional cholecystectomy and more efficient than alternative procedures like extracorporeal lithotripsy. Complication rates that are comparable to those of conventional cholecystectomy or even lower can be obtained, but since inexperience with the laparoscopic technique seems to be the main reason for complications, appropriate training of all surgeons must be provided to maintain and improve low morbidity rates.

### REFERENCES

1. Trede, M. and Schaupp, W. (1990) Ein Plädoyer für die Cholecystektomie—"Gold-Standard" der Gallensteintherapie. *Chirurg.*, **61**, 365–369.

2. Gilliland, T. M. and Traverso, L. W. (1990) Modern Standards for Comparison of Cholecystectomy with Alternative Treatments for Symptomatic Cholelithiasis with Emphasis on Long Term Relief of Symptoms. *Surg. Gynecol. Obstet.*, **170**, 39–44.
3. McSherry, C. K. (1989) Cholecystectomy: The Gold Standard. *Am. J. Surg.*, **158**, 174–178.
4. Bailey, R. W. and Zucker, K. A., Flowers, J. L., Scovill, W. A., Graham, S. M. and Imbembo, A. L. (1991) Laparoscopic Cholecystectomy—Experience with 375 Consecutive Patients. *Ann. Surg.*, **214**, 531–541.
5. Graves, H. A., Ballinger, J. F. and Anderson, W. J. (1991) Appraisal of Laparoscopic Cholecystectomy. *Ann. Surg.*, **213**, 655–664.
6. Southern Surgeons Club (1991) A Prospective Analysis of 1518 Laparoscopic Cholecystectomies. *N. Eng. J. Med.*, **324**, 1073–1078.
7. Frazee, R. C. and Roberts, J. W., Okeson, G. C., Symmonds, R. E., Snyder, S. K., Hendricks, J. C., and Smith, R. W. (1991) Open Versus Laparoscopic Cholecystectomy—A Comparison of Post-operative Pulmonary Function *Ann. Surg.*, **213**, 651–654.
8. Blatner, M. E., Wittgen, C. M., Andrus, C. H. and Kaminski, D. L. (1991) Cystic Duct Cholangiography During Laparoscopic Cholecystectomy. *Arch Surg.*, **126**, 646–649.
9. Sackier, J. M., Berci, G., Phillips, E., Carroll, B., Shapiro, S. and Paz-Partlow, M. (1991) The Role of Cholangiography in Laparoscopic Cholecystectomy. *Arch. Surg.*, **126**, 1021–1026.
10. Stoker, M. E., Leveillee, R. J., McCann, Jr J. C. and Maini, B. S. (1991) Laparoscopic common bile duct exploration. *Laparoend. Surg.*, **1**, 287–293.
11. Quattlebaum, J. K. and Flanders, H. D. (1991) Laparoscopic treatment of common duct stones. *Surg. Lap. Endo.*, **1**, 26–32.
12. Hunter, J. G. (1991) Avoidance of Bile Duct Injury During Laparoscopic Cholecystectomy. *Am. J. Surg.*, **162**, 71–76.
13. Petelin, J. B. (1991) Laparoscopic approach to common duct pathology. *Surg. Lap. Endo.*, **1**, 33–41.
14. Carroll, B. J., Phillips, E. H., Daykhovsky, L. and Grundfest, W. S. (1992) Laparoscopic choledochoscopy: An effective approach to the common duct. *J. Laparoend. Surg.*, **2**, 15–21.
15. Fabre, J. M., Przemyslaw, P., De Seguin des Hons, C., Lepage, B., Balmes, M., Baumel, H. and Domergue, J. (1992) Evaluation of the Laparoscopic Cholecystectomy on Patients with Simple and Complicated Cholecystolithiasis. *World J. Surg.*, **16**, 113–117.
16. Schirmer, B. D., Edge, S. B., Dix, J., Hyser, M. J., Hanks, J. B. and Jones, R. S. (1991) Laparoscopic Cholecystectomy—Treatment of Choice for Symptomatic Cholelithiasis. *Ann. Surg.*, **213**, 665–677.

